# Age-Related Differences in Motor Coordination during Simultaneous Leg Flexion and Finger Extension: Influence of Temporal Pressure

**DOI:** 10.1371/journal.pone.0083064

**Published:** 2013-12-10

**Authors:** Tarek Hussein, Eric Yiou, Jacques Larue

**Affiliations:** Laboratory CIAMS (Complexité, Innovation, Activités Motrices et Sportives), Team RIME (Risque, Intervention, Mouvement, Equilibre), University of Paris-Sud, Orsay, France; University of Ottawa, Canada

## Abstract

Although the effect of temporal pressure on spatio-temporal aspects of motor coordination and posture is well established in young adults, there is a clear lack of data on elderly subjects. This work examined the aging-related effects of temporal pressure on movement synchronization and dynamic stability. Sixteen young and eleven elderly subjects performed series of simultaneous rapid leg flexions in an erect posture paired with ipsilateral index-finger extensions, minimizing the difference between heel and finger movement onsets. This task was repeated ten times under two temporal conditions (self-initiated [SI] vs. reaction-time [RT]). Results showed that, first, temporal pressure modified movement synchronization; the finger extension preceded swing heel-off in RT, and inversely in SI. Synchronization error and associated standard deviation were significantly greater in elderly than in young adults in SI only, i.e. in the condition where proprioception is thought to be crucial for temporal coordination. Secondly, both groups developed a significantly shorter mediolateral (ML) anticipatory postural adjustment duration in RT (high temporal pressure) than in SI. In both groups, this shortening was compensated by an increase in the anticipatory peak of centre-of-gravity (CoG) acceleration towards the stance-leg so that ML dynamic stability at foot-off, quantified with the “extrapolated centre-of-mass”, remained unchanged across temporal conditions. This increased CoG acceleration was associated with an increased anticipatory peak of ML centre-of-pressure shift towards the swing-leg in young adults only. This suggested that the ability to accelerate the CoG with the centre-of-pressure shift was degraded in elderly, probably due to weakness in the lower limb muscles. Dynamic stability at foot-off was also degraded in elderly, with a consequent increased risk of ML imbalance and falling. The present study provides new insights into the ability of elderly adults to deal with temporal pressure constraints in adapting whole-body coordination of postural and focal components of paired movement.

## Introduction

Temporal pressure is known to be a constraint that strongly influences both the spatial and temporal aspects of motor coordination (defined as the temporal relationship between motor events) [1], [2], [3], [4], [5], [6]. High temporal pressure occurs when subjects are required to respond as rapidly as possible after a “go-signal”; this is called a reaction-time (RT) condition. On the contrary, low temporal pressure occurs when subjects do not face any go-signal and are instructed to take as much time as they need to prepare the movement, and to initiate it only when they feel ready; this is called a self-initiated (SI) condition. Motor coordination is required when the subjects have to trigger two distant effectors simultaneously. Then, a short latency between the two movement onsets (called “inter-onset latency”, IOL) systematically occurs even though the subjects report simultaneous onsets [1], [2], [3], [4], [5], [6]. Most importantly, IOL has been shown to depend on whether the subjects perform in an SI or TR condition. Classically, during simultaneous index-finger extension and single heel raising from a seated posture, finger movement onset systematically precedes heel movement onset in the RT condition (finger precedes heel), whereas the precession is reversed in the SI condition (heel precedes finger) [1], [2], [3], [4], [6]. A similar result was obtained during simultaneous jaw raising or lowering paired with heel raising or lowering [5]. All these studies interpreted these findings based on the Paillard’s model of temporal coordination [1], [2]. According to this model, finger (or jaw) precession over heel movement onset in the RT condition reflects the difference in the conduction time of the descending pathways, as if the two motor commands were simultaneously released through a common triggering signal in the motor cortex. In contrast, precession of the heel over the finger (or the jaw) in the SI condition suggests that the two motor commands are released by the motor cortex in such a way that the proprioceptive inflow associated with the production of each movement arrives at the same time at the central level, very likely at the cerebellar level [2], a structure often considered to be implicated in the evaluation of movement timing [4,7]. In this scenario, the motor command for the heel has to be released before the motor command for the finger (or jaw) because of the difference in the conduction time of the ascending pathways to the cerebellum. The proprioceptive inflow required for movement synchronization very likely originates from spindles located in the muscles that accelerate the focal limb [5], [8]. 

Proprioceptive inflow is known to be altered with aging, with a decrease in the quality and quantity of afferent inputs conducted to the central nervous system [9,10]. Therefore, the temporal coordination of simultaneous movements might be altered with aging, thus increasing IOL values and decreasing synchronization stability (corresponding to the trial-to-trial variability) as compared to young adults. This degradation is expected to be more acute in an SI condition where proprioception is critical for movement synchronization. To date, the influence of aging on the temporal coordination of simultaneous movements has not been addressed in the literature. Previous studies emphasized that an increased IOL during paired movements might be a source of degraded global motor performance, as revealed in studies in young adults [11], [12] [13], and patients with Parkinson’s disease [14]. Hence, besides the well-known slowness of isolated movements reported in the elderly population, it could be envisaged that, with aging, an increased IOL during paired movements contributes to decreased global motor performance. 

It is also worth noting that, in all of these studies [1], [2], [3], [4], [5], [6], the subjects performed very simple (i.e. single joint) movements involving body segments with low inertia such as the jaw, foot or index finger. However, paired movements involving the whole body also play an important role in normal motor behaviours. Additionally, these movements were systematically performed in a seated position, which is known to be highly stable. Therefore, these movements do not constitute a threat to body balance and, consequently, do not require the development of substantial “anticipatory postural adjustments” (APA). APA have been described as dynamic and electromyographic phenomena in the postural limbs resulting from motor commands released *before* the onset of the focal movement. APA act to counter in advance the disturbance to posture and balance elicited by the voluntary movement [15], [16]. The question remains how the central nervous system organizes temporal coordination between the focal and postural components of two simultaneous movements when one (or both) of these movements requires the development of substantial APA. 

Recent results are related to temporal coordination between various paired movements involving the whole body and requiring large APA, e.g. a lunge paired with a touche in fencing [11], [12], [13], arm raising or pointing paired with step initiation [17], [18], [19], and arm pointing paired with leg flexion [20]. In these studies, movement synchronization was either imposed by the experimenter (e.g. the touche had to be triggered before the swing heel-off for lunging, as recommended in fencing rules) or a single mode of movement triggering (RT or SI) was employed. Consequently, the question of whether Paillard’s model of temporal coordination might be extended to paired movements involving the whole body could not be addressed in these studies. Hence, the first goal of the present study was to test whether Paillard’s model of temporal coordination might be extended to paired movements involving the whole body and, more specifically, whether aging affects this coordination.

Besides its effect on synchronization, temporal pressure is known to influence both spatial and temporal aspects of coordination between posture and movement. Previous studies reported that, in healthy young adults, the duration of APA associated with various motor tasks, including isolated leg flexion [21], gait initiation [22] or upper limb raising [23], [24], was shortened in an RT condition as compared to an SI condition. During rapid leg flexion in a standing posture, it was shown that young adults were able to compensate for this shortened APA duration through increased APA amplitude (in terms of a peak of lateral centre of pressure shift) so that the lateral centre of gravity dynamics at the time of swing foot-off remained unchanged [21]. This adaptation of lateral dynamics to temporal pressure allowed the subjects to hasten voluntary movement onset and to maintain postural stability in the final (unipodal) posture. Because it is known that most falls in the elderly occur laterally due to deficits in lateral stability control [25], the question might be addressed as to whether the elderly are able to develop a similar adaptive postural behaviour as young adults when exposed to temporal pressure constraints. Hence, the second goal of this study was to investigate the effect of aging on the relationship between temporal pressure and APA.

Thus, the present work examined the aging-related effects of temporal pressure on the two above-reported aspects of motor coordination, synchronization between two voluntary movements and coordination between posture and movement. Young adults and healthy elderly participants performed series of simultaneous rapid leg flexions paired with ipsilateral index-finger extensions in the erect posture in both an RT and an SI condition. Leg flexions are known to involve large APA (lasting approximately 400 milliseconds [20,21]) while index extensions involve virtually no APA. The following three hypotheses were tested: 1) in the RT condition, the motor commands associated with each focal movement (finger extension and heel raise for leg flexion) are released simultaneously at the cortical level. Because leg inertia is much greater than finger inertia and because the efferent pathway to the swing foot is longer than to the finger, there should be a precession of finger extension onset over voluntary leg flexion onset (as dated with the swing heel-off time). In this condition, there should not be any change in IOL in the elderly as compared to young adults, at least insofar as body height (and thus the efferent pathway lengths to the mobile foot and finger) remains equivalent across these two groups; 2) In the SI condition, the temporal ordering of focal movement onsets is reversed (heel precedes finger). IOL and the variability of IOL (estimated with the standard deviation) should be greater in the elderly as compared to young adults because the proprioceptive inflow required for temporal coordination is altered; and 3) The ability to adapt spatial and/or temporal features of APA to temporal pressure is degraded with aging.

## Materials and Methods

**Figure 1:**
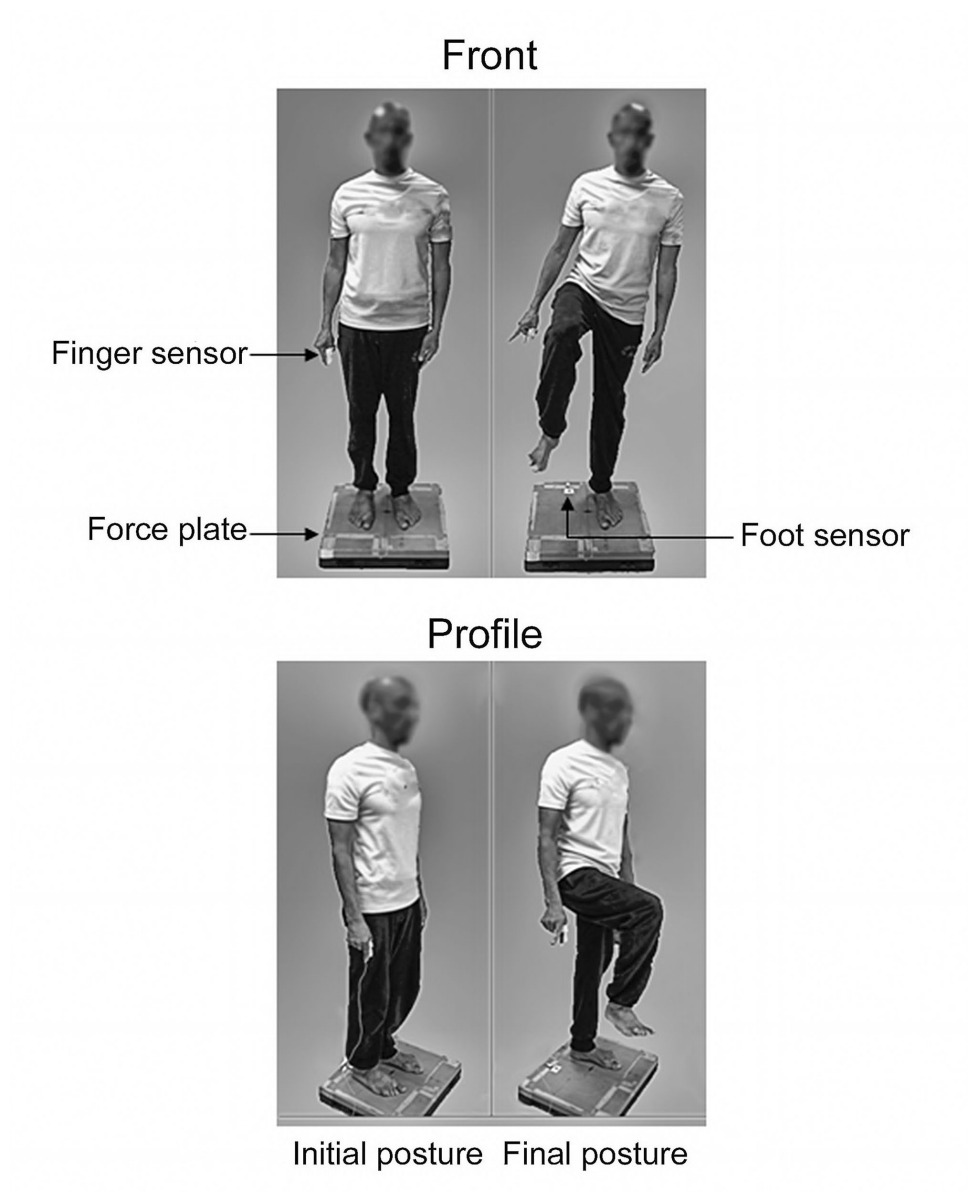
Initial/final posture and experimental set-up. Upper panels: front views; Lower panels: profile views. The subject of the photograph has given written informed consent, as outlined in the PLOS consent form, to publication of the photograph.

The subject of the photograph (Figure 1) has given written informed consent, as outlined in the PLOS consent form, to publication of the photograph.

### Participants

Sixteen young adults (seven males/nine females, right handed, 27 ± 7 years old [mean ± 1 standard deviation], height 170 ± 9 cm, body mass 72 ± 13 kg) and eleven elderly adults (four males/seven females, right handed, 80 ± 8 years old, height 169 ± 4 cm, body mass 71 ± 8 kg), free of any known neuromuscular disorders, took part in the present experiment. All participants gave written consent after being informed as to the nature and purpose of the experiment which was approved by the ethics committee of the Hospital Centre of Longjumeau (France) who specifically approved this study. The study conformed to the standards set by the Declaration of Helsinki. There was no significant difference in height or body mass between the two groups (p=0.81). Elderly participants were excluded if they had any history of serious orthopaedic, cardiovascular, or neurological diseases or dysfunctions, any history of falls, or any pain at the time of the experiment. The elderly subjects were required to undergo a brief clinical examination to evaluate their motor control ability (using the Berg Balance Scale [BBS] and the Timed Up and Go test [TUG]) and cognitive functions (with the Mini-Mental State Examination [MMSE]). On average, the score obtained in these tests showed that they had basic mobility skills within normal limits for frail elderly subjects (TUG score = 12 ± 3 seconds), with a low fall risk (BBS score = 52 ± 4 [total score = 56]), and no cognitive problems or temporo-spatial disorientation (MSSE score > 26 [total score = 30]).

### Experimental procedure

#### Main experiment

The participants performed series of ten leg flexions in an erect posture with the dominant (right) leg, in isolation (isolated condition) or in combination with an extension of the index finger of the ipsilateral hand (synchronized condition; Figure 1). In the synchronized condition, leg raising and finger extension had to be performed simultaneously. The subjects were required to perform the movements at maximal velocity in both the RT condition (condition with high temporal pressure) and the SI condition (condition with low temporal pressure). So, each subject was tested under four conditions, for a total of 40 trials. Because adding the finger movement to leg flexion in the synchronized conditions did not modify leg flexion dynamics (as revealed through a comparison with the isolated condition), the results reported hereafter will be limited to the paired movements. The young and elderly adults were randomly assigned to two subgroups to counterbalance the order of the conditions. Half of the subjects began with the RT condition followed by the SI condition and conversely for the other half of the subjects. In the RT condition, the participants had to initiate the paired movements as soon as possible following an acoustic signal delivered by the experimenter. The acoustic signal was given by a buzzer controlled by a custom-made program written in Matlab™ (R11) running on a PC. In the SI condition, the participants were told to initiate the movements when they felt ready after receiving a “all set” signal; it was made clear to the participants that the “all set” signal was not a “go” signal and that they could take as much time they needed to prepare their movements and to perform the movement only when they felt ready. 

In both conditions, the participants stood barefoot and upright with their arms alongside their body, their feet shoulder-width apart, and with their gaze directed forward to a small target at eye level (2 cm diameter, 3 m distant). The feet position in the initial posture was marked on millimetric paper. The experimenters asked the participants to systematically position their feet inside these marks. In the final posture, the participants stood on one foot, with the lower leg flexed approximately 90° with the thigh and the thigh flexed approximately 90° with the trunk (Figure 1). This posture had to be maintained for approximately three seconds before the participants could return to the initial posture. The elderly participants wore a seat harness (model 40, manufactured by Liko, maximum weight 200 kg) affixed to a ceiling rail system (rail Liko™) with an engine (Likorall 242 ) in order to prevent any contact with the ground in case they lost their balance. The participants were repeatedly reminded of the instructions regarding the task constraints 2 familiarization trials were run in each condition (not recorded). Ten minutes of rest between each condition and 20 seconds of rest between trials were imposed to avoid the effects of fatigue. 

#### Pilot experiment

In this pilot experiment, we wanted to compare the error of synchronization produced in the main experiment described above, with the error of synchronization produced during the simultaneous single foot extension with index-finger extension in the seated posture, corresponding to the paired movements classically employed in the literature [1], [2], [3], [4], [6]. The length of the efferent pathways for triggering the voluntary movements are similar in these two paired movements since, in both cases, the swing heel raise and the finger extension correspond to the onset of the voluntary movements. The difference in the error of synchronization between these two paired movements could therefore be ascribed mostly to the difference of inertia between just the foot (pilot experiment) and the whole leg which supported half of the body weight (main experiment). This pilot study involved only healthy young participants (N=16). These participants were the same as those recruited for the main experiment. The protocol was exactly the same as for the main experiment (and the same as in [4]) except that the participants sat comfortably on a chair with their arms hanging freely alongside their trunk. The participants had to perform series of ten simultaneous single heel raises from the ground with ipsilateral index-extension in a RT and SI condition. 

### Data recordings

In both the main and pilot experiment, the movements were performed on a force-plate (AMTI, 60*60 cm, OR-6). Only the kinetic data related to the main experiment will be reported in the present study. Mediolateral (ML) centre of gravity acceleration was obtained from the ground reaction forces according to Newton’s second law. ML centre of gravity velocity and position were obtained through successive integrations of the acceleration signal [21], [26], [27], [28]. The centre of pressure position along the ML direction (yP) was obtained with the ratio [moment along the anteroposterior axis/vertical ground reaction force]. To detect the onset of swing heel-off in both the main and the pilot experiment (this instant corresponded to the onset of the voluntary lower limb movement in both paired movements [21], [26], [27], [28]), the heel of the swing leg made contact with a metal plate connected to a custom-made circuit; a simple disruption of the contact between the skin and the plate produced a clear Transistor-Transistor-Logic (TTL) signal, varying in an all-or-nothing fashion from 0 to 5 Volts (0 Volt corresponded to the output when the skin was in contact with the plate; 5 Volts corresponded to the output when that contact was broken, i.e. when the swing heel left the ground). This system provided an instantaneous signal avoiding the mechanical delays found in conventional switches. The participants grasped a similar system between the index and the thumb of the ipsilateral hand to detect the onset of voluntary finger extension (Figure 1). Both of the TTL signals (from the fingers and the swing heel) were recorded simultaneously on a PC equipped with an A/D card (Computer Boards TM, model CIO-DAS1602) with a time resolution set at 1 ms. Data acquisition and stimulus display were controlled by a custom-made program written in Matlab^TM^ (R11). Force-plate signals were recorded with a sampling rate of 250 Hz. Signals from the switches were sampled at 1000 Hz. 

### Experimental variables

#### Biomechanical variables

All durations were computed relative to “t0” which corresponded to the point in time where yP level exceeded the “background” yP mean level ± 2 standard deviations in the quiet standing posture. The mean “background” yP activity was obtained by averaging the yP trace (over a 200 ms time window) during the quiet standing period that preceded the onset of the acoustic signal. Therefore, the duration of APA corresponded to the interval between t0 and the swing heel-off. The unloading phase duration of the swing leg was measured by the difference in time between swing heel-off and swing foot-off. The amplitude of the ML APA was estimated with the peak deviation of the yP trace and the peak deviation of the ML centre of gravity acceleration trace during the APA time window (y’’G_APA_). ML postural stability at the swing foot-off time was quantified with the following two variables: the ML CoG velocity (y’G_FO_) and the ML “gap” between the centre of gravity (yG_FO_) and the centre of pressure (yP_FO_) at the swing foot-off time (STAB1), with STAB1=yP_FO_-yG_FO_. A perfectly stable postural state along the ML direction is reached at foot-off time if these two quantities are simultaneously zero. The results reported hereafter, however, show that this case never occurred. As in our previous studies [21], [28], we therefore computed an indicator of dynamic stability at the swing foot-off time (termed “STAB2”), which corresponded to the ML gap between a quantity termed “extrapolated centre of mass” (YcoM, [29]) and the centre of pressure (yP_FO_) at the swing foot-off time. Similar to STAB1, STAB2=yP_FO_-YcoM_FO_ (as defined by Hof et al. [29]), with YcoM=yG+y’G/ω0, where ω0=√g/L is the eigenfrequency of the body modelled as an inverted pendulum of length L, L=H*0.575 (H is the body height) and g is the acceleration of gravity=9.81 m/s^2^. YcoM_FO_ can be defined as an extrapolation of the centre of gravity in the direction of its velocity [29]. In order to maintain dynamic stability, the YcoM position at foot-off should be within the base of support, i.e. within the stance foot in the present study. Therefore, it will be considered that the greater the STAB2 (in absolute value), the further the participant is from this condition for dynamic stability. Finally, the peak velocity of the centre of gravity along the vertical axis was taken as an indicator of the performance of leg raising (focal performance; [20], [21], [28]). 

#### Synchronization-related variable

Inter-onset latency (IOL) was used as an indicator of synchronization error; it was the latency between heel raise onset (t1) and finger extension onset (t2) (IOL = t1-t2). Thus, a perfect synchronization produced an IOL=0, while a negative IOL indicated that swing heel-off onset preceded finger extension onset (heel then finger), and conversely for a positive IOL (finger then heel). The standard deviation of IOL was taken as an indicator of synchronization stability.

### Statistics

In the main experiment, individual means and standard deviations were calculated for both the biomechanical and synchronization-related variables. A [2 (Age) X ^2^ (temporal pressure constraint)] ANOVA with repeated measures on the last factor was used to test the differences between the means of biomechanical-related variables. In the pilot experiment, only mean values and standard deviations of synchronization-related variables were calculated. A [2 (postural condition) X ^2^ (temporal pressure constraint)] ANOVA with repeated measures was used to test the difference of synchronization-related variables between the pilot and the main experiment (healthy young adults only). The alpha level was set at 0.05. A Tukey *post hoc* test was used when significant interactions were found. Moreover, planned comparisons were used to test specific hypotheses 1 and 2 (see introduction), which solely included the synchronization-related variables obtained in the main experiment. The normality of data was checked with the Kolmogorv-Smirnov’s test and the homogeneity of variances was checked with Barltett’s test prior to performing the different ANOVAs.

## Results

### Description of the biomechanical traces

**Figure 2:**
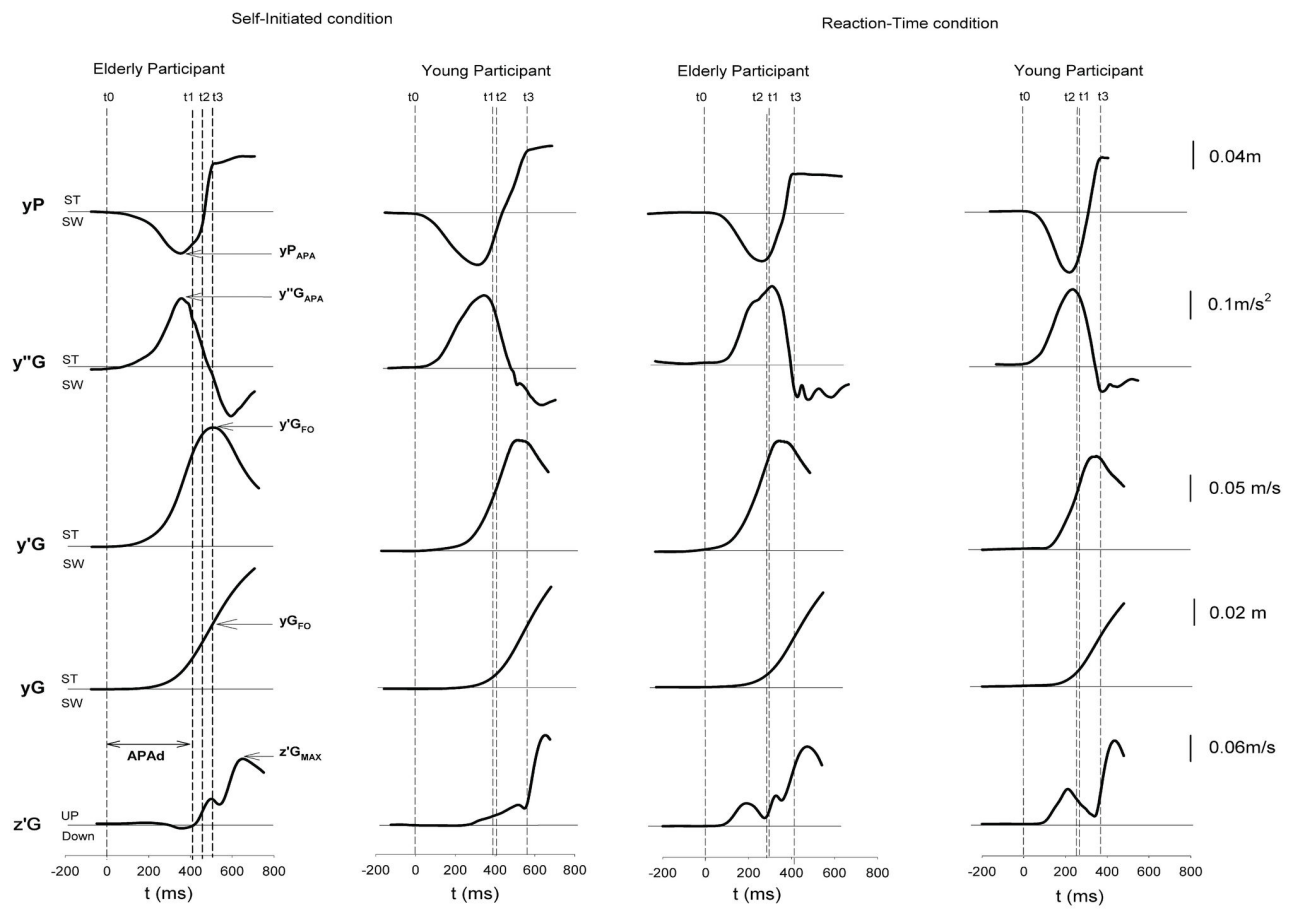
Example of biomechanical traces during paired leg flexion with index-finger extension in the reaction-time and self-initiated conditions (one trial in one representative subject in the young and elderly adults group). yP, y”G, y’G, yG, z’G: mediolateral (ML) centre of pressure (CoP) displacement, ML centre of gravity (CoG) acceleration, ML CoG velocity, ML CoG displacement and vertical CoG velocity, respectively. t0, t1, t2, t3: onset variation of yP trace from baseline, swing heel-off, index-finger extension (hand response) and swing foot-off time, respectively. Sw, St, Up, Down: swing leg side, stance leg side, upward and downward, respectively. APAd, yP_APA_, y’’G_MAX_, y’G_FO_, yG_FO_, z’G_MAX_: APA duration, peak of ML CoP displacement during APA, peak of ML CoG acceleration, ML CoG velocity and displacement at the foot-off time and peak of vertical CoG velocity, respectively.

In the RT and SI conditions, swing heel-off was systematically preceded by dynamic phenomena corresponding to APA (Figure 2). During these APA, the trace of the ML centre of gravity acceleration reached a peak value towards the stance leg side while the ML centre of pressure trace reached a peak value towards the swing leg side (these two traces mirrored each other). The trace of the ML centre of gravity velocity reached a peak value towards the stance leg side at approximately swing foot-off time. The ML centre of gravity continuously moved towards the stance leg side. The trace of vertical centre of gravity velocity reached a peak value following swing foot-off time. The time course of these biomechanical patterns was roughly similar in the young and elderly adults. 

### The influence of temporal pressure and aging on the synchronization between swing heel-off and index-finger extension

#### Pilot experiment

**Figure 3:**
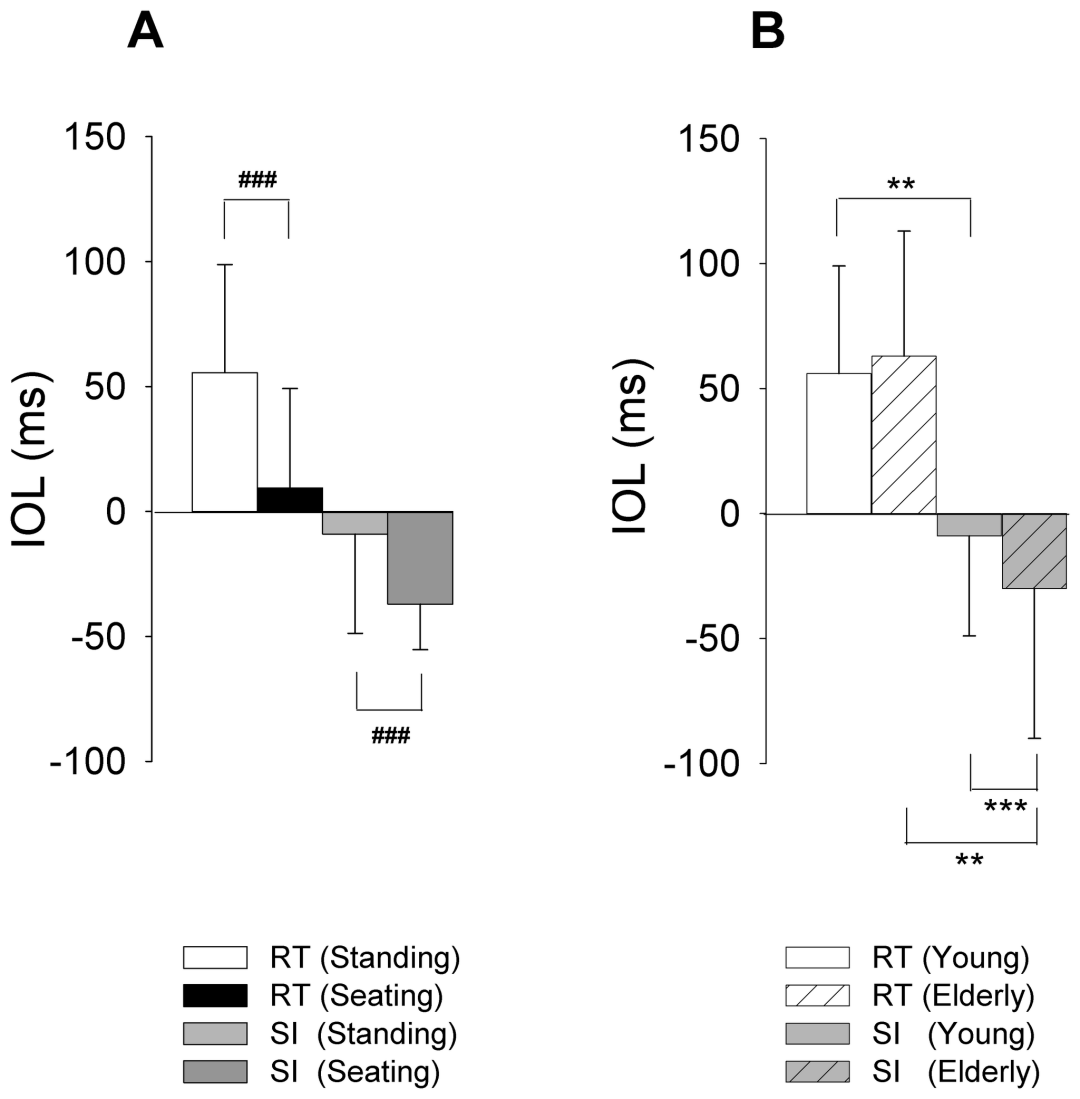
Comparison of simultaneous index-finger extension and swing heel-off inter-onset latency (IOL) in the pilot (A) and main (B) experiments. RT, SI: reaction-time and self-initiated condition, respectively. Positive IOL indicates a precession of index-finger extension over swing heel-off. Negative IOL indicates a precession of swing-heel off over index-finger extension. Reported values are mean ± 1 standard deviation (all participants combined). RT: reaction-time condition; SI: self-initiated condition. ###: significant main effect of posture with p < 0.001; **, ***: significant difference with p < 0.01 and p < 0.001, respectively, as revealed with the planned comparisons. Note the inversion of the IOL values between the RT and SI conditions in the Figures A and B.

In this experiment, we compared the IOL in standing and seated postures in the young adults group only. There was a significant main effect of the posture on the IOL (F_(1,15)_ = 41.15, p < 0.001; Figure 3A). The IOL was significantly different in the standing position (M= 23 ± 40 ms; mean of raw IOL values obtained in the SI and RT conditions confounded) and the seated position (M= -14 ± 38 ms). There was no significant interaction (F_(1,15)_ = 0.40; p=0.53). However it is important to note in the Figure 3A that the performance changed in opposite direction according to the temporal constraint. Indeed, in the RT condition, swing-heel off occurred after the index-finger extension. However, the swing-heel off preceded the index-finger extension in the SI condition. Therefore, the heel-finger synchronization was significantly better in the seating posture when the subjects had to react as fast as possible; whereas, it was significantly better in the standing posture when the subjects self initiated their movements.

#### Main experiment

Planned comparisons were applied to test the specific hypothesis. As expected, elderly group produced significantly larger IOL (F_(1,25)_ = 10.48, p < 0.001) and associated standard deviation (F_(1,25)_ = 39.18, p < 0.001) than the young adults group in the SI condition, but not in the RT condition (p > 0.05). Planned comparison further showed significantly different IOL values in the RT and in the SI conditions in both the young adults (F_(1, 15)_ = 10.60; p < 0.01) and the elderly group (F_(1,10)_ = 20.75; p < 0.01). 

In the RT condition, the onset of index extension preceded swing heel-off onset (finger then heel) with an IOL = 55 ± 43 ms in the young adults group and with an IOL = 63 ± 49 ms in the elderly group (Figure 3B). In the SI condition, this temporal ordering was reversed, i.e. the swing heel-off onset preceded index extension onset (heel then finger) with an IOL = -9 ± 30 ms in the young adults group and with an IOL = -30 ± 60 ms in the elderly group.

### The influence of temporal pressure and aging on the biomechanical organization of leg flexion

#### Initial posture

There was a significant effect of temporal pressure on the initial position of the centre of pressure (F_(1,25)_ = 12.24, p < 0.01). Specifically, the centre of pressure was located more towards the forthcoming stance leg in the RT condition than in the SI condition in both the elderly and the young adults group. 

#### Spatio-temporal APA features

**Figure 4:**
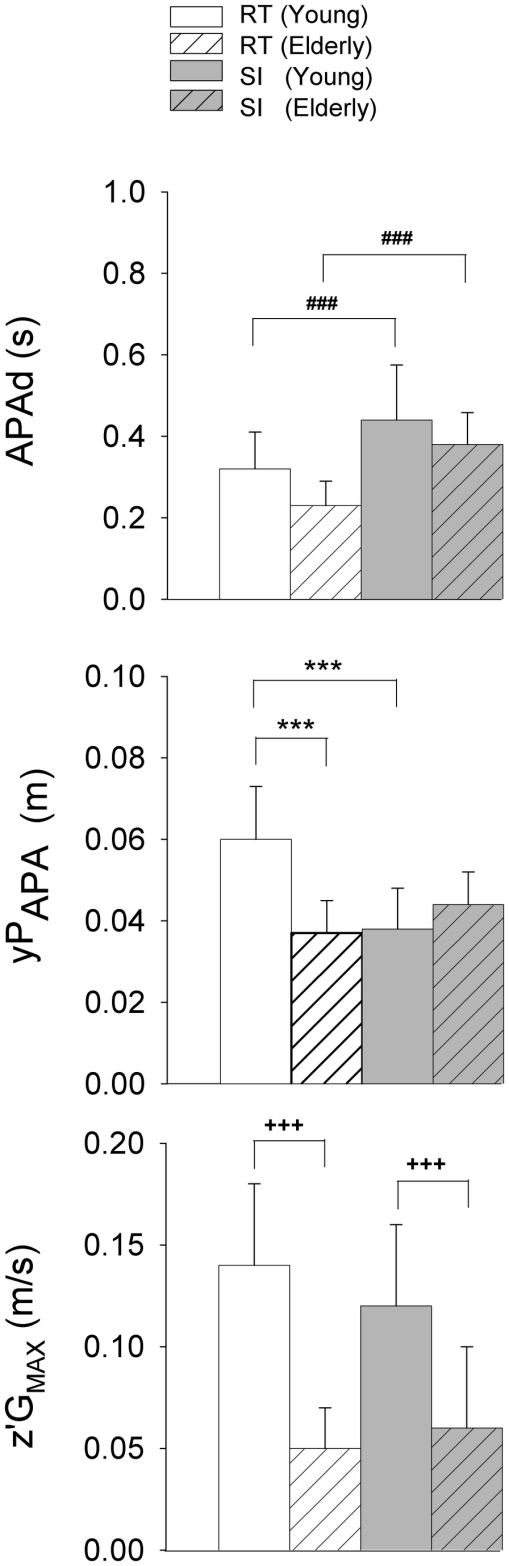
Comparison of selected spatio-temporal features of APA and focal movement performance across groups (young vs. elderly adults) and conditions (self-initiated vs. reaction-time). APAd, yP_APA_, z”G_MAX_: duration of APA, peak of mediolateral centre of pressure displacement towards swing leg side, peak of vertical centre of gravity velocity, respectively. Reported values are mean ± 1 standard deviation (all participants combined). RT: reaction-time condition; SI: self-initiated condition. ###: significant main effect of time pressure with p < 0.001; ***: significant difference with p < 0.001 as revealed with the *post hoc* test; +++: significant main age effect with p < 0.001.

There was a significant main effect of temporal pressure on APA duration (F_(1,25)_ = 32.40, p < 0.001) which was significantly shorter in the RT condition than in the SI condition in the young adults (p < 0.001) and the elderly group (Figure 4). Age effect on APA duration did not reach significance, though this variable tended to be lower in the elderly adults than in the young adults (F_(1,25)_ = 3.33, p = 0.08). Interestingly, there was a significant age-to-temporal-pressure interaction for the peak of anticipatory centre of pressure displacement toward the swing leg side (F_(1,25)_ = 30.43, p < 0.001). Tukey post hoc test showed that this peak was significantly greater in the RT as compared to the SI condition for the young adults group (p < 0.001), whereas the difference was not significant for the elderly adults (p > 0.05). For the elderly, this peak even tended to reach a greater value in the SI condition (4.4 cm) than in the RT condition (3.7 cm). In addition, this peak was significantly greater in the young adults than in the elderly group in the RT condition (p < 0.001; Figure 4).

There was a main effect of time pressure on the peak of anticipatory centre of gravity acceleration towards the stance leg side (F_(1,25)_ = 15.12, p < 0.001), with a greater peak value in the RT than in the SI condition for both age group. In addition, there was a significant main effect of age for this variable (F_(1,25)_ = 7.25, p < 0.05), with the elderly adults having a greater peak value than the young adults. Finally, there was no significant effect of either age or temporal pressure on the duration of the unloading phase (p > 0.05).

#### Focal performance

There was a significant age effect (F_(1,25)_ = 28.50, p < 0.001) for the peak of vertical velocity of the centre of gravity. The young adults produced significantly greater peaks (0.13 ± 0.01 m/s) than did the elderly (0.06 ± 0.01 m/s) (Figure 4). Interestingly, the peaks produced by the elderly were similar for both temporal constraints; however, the young adults produced higher peaks in the RT condition in comparison with the SI condition, but this difference just failed to reach statistical significance (p=0.06). 

#### Postural dynamics at swing foot-off time

**Figure 5:**
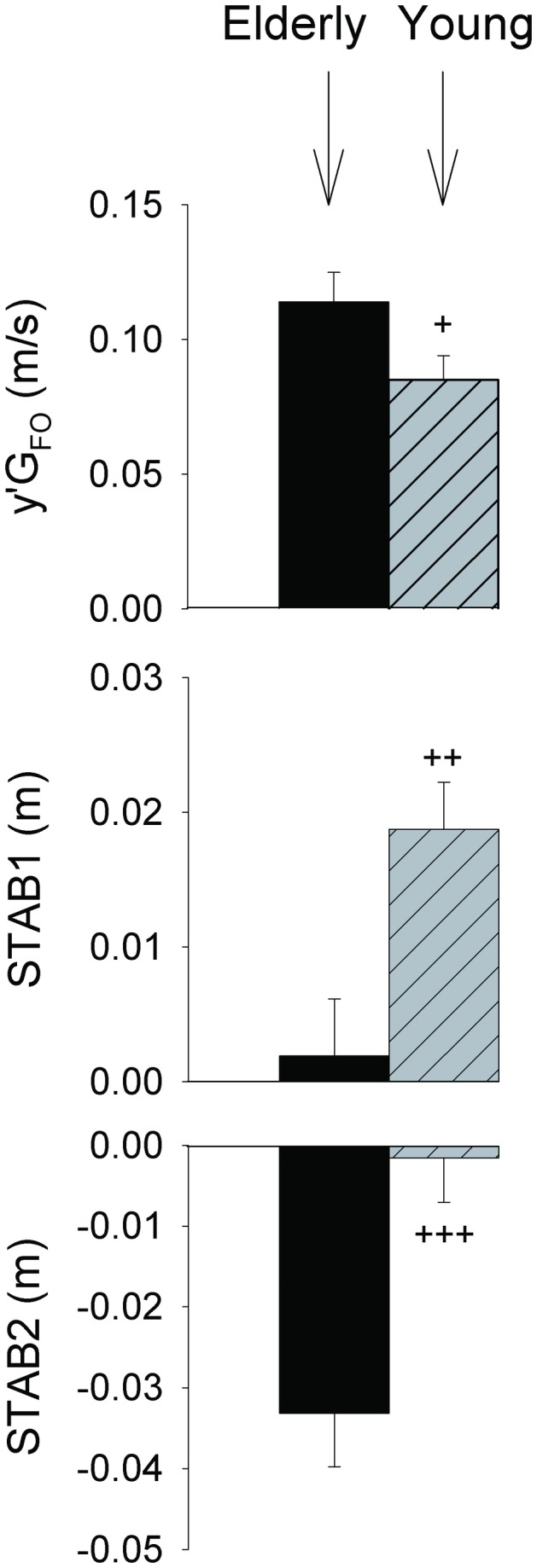
Comparison of postural dynamics at swing foot-off across groups (young vs. elderly adults). Only statistics related to the age effect are reported in this figure since there was no significant effect of temporal pressure on the reported variables. STAB1, STAB2, y’G_FO_: ML “gap” between the centre of gravity and centre of pressure position at swing foot-off, ML “gap” between the extrapolated centre of mass and centre of pressure position at swing foot-off, ML centre of gravity velocity at swing foot-off, respectively. STAB1=0 indicates that the ML coordinate of the centre of gravity and the centre of pressure are aligned vertically at swing foot-off (only the ML direction is considered in this study). Positive STAB1 value indicates that the centre of gravity has not yet passed above the centre of pressure. STAB2=0 indicates that the extrapolated centre of mass and centre of pressure are aligned vertically. Negative STAB2 value indicates that the extrapolated centre of mass is located beyond the centre of pressure (towards the external side of the stance leg). Reported values are mean ± 1 standard deviation (all participants combined). +, ++, +++: significant main age effect with p < 0.05, p < 0.01 and p < 0.001, respectively.

There was a significant effect of age but not of temporal pressure on the following variables: ML velocity of the centre of gravity at swing foot-off time (F_(1,25)_ = 4.07, p < 0.05), ML “gap” between the centre of pressure and the centre of gravity (STAB1 = yP_FO_-yG_FO_, F_(1,25)_ = 9.37, p < 0.01), and ML gap between the centre of pressure and the extrapolated centre of mass (STAB2 = yP_FO_-YcoM_FO_, F_(1,25)_ = 13.61, p < 0.001). Specifically, STAB1 was greater in the young adults than in the elderly group (p < 0.01, Figure 5). In the elderly group, STAB1 was virtually zero (i.e. the centre of pressure and the centre of gravity were almost aligned vertically at swing foot-off time) while it was positive in the young adults group (i.e. the centre of gravity had not yet passed above the centre of pressure). At this time, the elderly reached a greater ML centre of gravity velocity than did the young adults (p < 0.05). This velocity was positive in both groups, i.e. it acted to propel the centre of gravity towards the external side of the stance foot. STAB2 was greater (in absolute value) in the elderly than in the young adults group (p < 0.001). STAB2 was virtually zero in the young adults group (i.e. the centre of pressure and the extrapolated centre of mass were almost aligned vertically), while it was negative in the elderly group (i.e. the extrapolated centre of mass was located beyond the centre of pressure towards the external side of the stance foot). 

## Discussion

This work examined the aging-related effects of temporal pressure on two aspects of motor coordination, i.e. synchronization between two voluntary movements, and coordination between posture and movement. In the following discussion, we first address the question of whether Paillard’s model of temporal coordination (which was elaborated on the basis of simple paired movements) might be extended to paired movements involving the whole body and, more specifically, whether aging affects this coordination. Second, we discuss the effect of temporal pressure on the biomechanical organisation of APA in young and older adults. Last, we discuss the influence of temporal pressure on postural dynamics at foot-off in young and elderly adults. 

### The influence of temporal pressure on the synchronization of leg flexion and finger extension in young and elderly adults

Recent literature reports results related to the synchronization of various paired movements involving large APA in healthy adults, e.g. a lunge paired with a “touche” in fencing [11], [12], [13], arm raising or pointing paired with step initiation [17], [18], [19], and arm pointing paired with leg flexion [20]. For example, during simultaneous leg flexion and arm pointing performed in an RT condition, Yiou [20] reported that the onset of the arm movement systematically occurred before the onset of voluntary leg flexion as marked with swing heel-off time, i.e. arm-then-heel synchronization was observed. Similarly, in the present study, finger extension systematically preceded swing heel-off time in the RT condition, i.e. finger-then-heel synchronization was observed. These results are in accordance with Paillard’s model of temporal coordination in a condition with high temporal pressure [1], [2]. According to this model, the motor commands for the two focal movements – finger extension and swing heel raising in the present study – would be sent simultaneously by the motor cortex. Because of the difference of length in the efferent pathway, finger extension preceded swing heel-off. 

A parameter that is not taken into account in Paillard’s model, and that may potentially influence temporal coordination, is the difference of inertia between the simultaneously mobilised focal limbs. This simplification in the model likely did not influence movement synchronization much because the difference of inertia between the focal limbs classically mobilised was relatively small [1], [2], [3], [4], [5], [6]. In contrast, in the present study, the inertia of the swing leg (supporting half of the body’s weight) was much greater than the inertia of the index finger. Consequently, the time required before a given focal limb can be moved following muscle excitation (i.e. electromechanical delay) is much greater for the whole leg than for the index finger. Thus, in addition to the difference of length in the efferent pathways controlling finger extension and swing heel raise, a large difference of inertia between the two focal limbs probably also contributed to increasing the error of movement synchronization. Accordingly, the mean IOL found in the present study (55 ms for healthy young subjects) was much greater than the mean IOL found during simultaneous foot extension in a seated posture paired with finger extension (9 ms in the pilot study of the present experiment). This 9 ms time is exactly the same as that found for healthy young subjects by Blouin et al. [4] in experimental conditions similar to the pilot study. The error of synchronization thus increased by 46 ms when the whole leg was mobilised rather than the foot alone. 

The increase in synchronization error due specifically to the greater inertia of the whole leg as compared to the foot alone can be theoretically estimated with the electromechanical delay between the onset activation of the prime mover muscle responsible for swing heel-off and the swing heel-off event. For both just-the-heel-raise in the seated posture and leg flexion in the erect posture, the *soleus* (ankle extensor) can be considered as being responsible for the swing heel-off event [30]. This electromechanical delay has been estimated at 25 ms for just-the-heel-raise in the seated posture [5] and 70 ms for the leg flexion in the erect posture [30]. The difference between these two delays (45 ms) thus fits with the 46 ms difference of IOL reported above. Therefore, it appears that, in the present study, most of the synchronization error could not be ascribed to the length of the different efferent pathways between the ankle and finger muscles (this difference can be estimated as contributing approximately 9 ms of the 55 ms IOL found in the present study), but to the difference of inertia between the focal limbs mobilised. This difference can be estimated as contributing approximately 46 ms of the 55 ms IOL. Now, beside the difference of inertia between the whole leg and the foot alone, it is also possible that the difference in movement complexity between just-the-heel-raise (which mainly involves the activation of the plantar flexors) and the leg flexion (which requires the coordinated activation of the plantar, knee and hip flexors) also contributed to the IOL difference.

Despite the relatively large error of synchronization reached by participants in the RT condition (up to 63 ms in the elderly), it is worthwhile noting that the 80 ms IOL threshold required to detect asynchrony between focal limb movement [1] was not reached, which explains why the subjects still reported simultaneous focal movement onsets. Thus, the present results suggest that, in conditions with high temporal pressure, the central nervous system may not take into account the difference of limb inertia for movement synchronization, at least as long as the error of synchronization does not exceed the 80 ms IOL threshold. On-going research is investigating whether IOL is increased when inertia is added at the ankle of the swing leg.

In regards to the SI condition, the present results agree with a recent study on motor coordination during simultaneous lateral arm raising paired with forward step initiation [19]. In this study, the authors reported that the onset of arm raising occurred after swing heel-off, i.e. heel-then-arm synchronization was observed. Similarly, in the present study, finger extension onset followed swing heel-off onset in the SI condition (heel-then-finger synchronization), thus contrasting with the finger-then-heel synchronization observed in the RT condition. These latter results are also in agreement with Paillard’s model of temporal coordination in a condition with low temporal pressure. According to this model, the focal movements are synchronized in such a way that the reafferent proprioceptive inputs arrive at the same time at the central level (very likely at the cerebellum [4,7]) thus establishing simultaneity of motor events at the perceptive level. In the present study, we found that IOL (9 ms in healthy young adults) was much smaller than IOL obtained during simultaneous just-heel-raise from the seated posture paired with finger extension (36 ms in the pilot study of the present experiment). This delay is very close to the 31 ms IOL found for healthy young subjects [4] in experimental conditions similar to the pilot study. Thus, the error of synchronization decreased by 27 ms when the whole leg was mobilised rather than just the foot. It is possible that the greater proprioceptive inflow elicited by the focal leg flexion (which requires ankle extension, and knee and hip flexion) as compared to a just-heel-raise (which requires only ankle extension), contributed to improving the precision of movement synchronization. 

The present results further showed that the temporal pressure constraint induced similar effects on movement synchronization in the elderly and in the healthy young participants, i.e. finger-then-heel and heel-then-finger synchronization was observed in the RT and SI condition, respectively. The ability to adequately synchronize the focal motor commands according to temporal pressure therefore seems preserved in the elderly. In other words, the proprioceptive inflow arising from the mobile limbs still seems to remain a sufficiently reliable source of information to synchronize focal movements. However, it was also observed that the elderly adults reached a significantly greater IOL (30 ms) than did the young adults (9 ms) in the SI condition only, i.e. the precision of movement synchronization was degraded. Note that this IOL lengthening was very small in amplitude (21 ms) and therefore may not contribute substantially to the decrease in performance during paired movements with aging (in terms of global movement duration). In the present study, the decrease in performance with aging was associated mainly with the decrease in the peak of leg flexion velocity (by approximately 50% as compared to young adults), rather than with a substantial alteration of movement synchronization. The results also showed that the standard deviation associated with the mean IOL was twice as great in the elderly (60 ms) than in the young adults (30 ms), i.e. the stability of movement synchronization was lower in older adults. Therefore, these results agree with hypotheses 1 and 2 stated in the Introduction, and provide some support for the important role played by proprioception - which is known to be altered with aging [9,10] - to finely control the precision and stability of movement synchronization. 

### The influence of temporal pressure on the biomechanical organisation of APA associated with rapid leg flexion in young and elderly adults

In young adults, the results showed that APA duration in the RT condition was significantly shortened by 28% as compared to the SI condition. Such an effect of temporal pressure on APA duration confirms recent data on the same task [21] (note that the number of participants was twice as great in the present study) and is congruent with data from the literature on gait initiation [22] and upper limb tasks [23], [24,31]. This shortening presumably reflects a strategy to hasten the onset of the voluntary movement (swing heel-off in the present study) and thus reduce the RT. This shortening, though quite large, did not modify the postural dynamics at the swing foot-off time, as revealed by the comparison of ML centre of gravity velocity, STAB1 and STAB2 variables across the temporal conditions. Hence, it seems that postural adaptation occurred in the condition with high temporal pressure to compensate for the large APA shortening. We discuss the possible nature of this adaptation. 

First, we observed that young adults leaned more laterally towards the stance leg in the initial standing posture in the RT vs. SI condition. This change may have facilitated body weight transfer during APA, as shown during lateral leg raising [32] or gait initiation [33]. However, the difference in the initial centre of pressure positioning between these two conditions, though significant, was very small in amplitude (0.3 cm) and probably may not be a major factor in postural adaptation. Second, it could be envisaged that an increase in the duration of the unloading phase (corresponding to the time between swing heel-off and swing foot-off) compensated for the shortening of APA duration (corresponding to the time between the onset of biomechanical traces t0 and swing heel-off). In this scenario, the time between t0 and the swing foot-off event would remain equivalent across conditions despite the observed APA duration shortening. However, the results showed that the duration of the unloading phase did not significantly change across the conditions. Last, and very likely most critically, we found that the peak of anticipatory ML centre of pressure displacement was greater in the RT than in the SI condition. This difference reached 2.5 cm, representing a 63% increase. According to the classical model of interaction between the centre of gravity and the centre of pressure [26], [27] the propulsive forces that accelerate the centre of gravity towards a given direction result from a “gap” between the centre of pressure and the centre of gravity. During APA associated with a lower limb task, such as lateral leg raising [34], gait initiation [19,26,27] or leg flexion [20,21], this gap is created by the centre of pressure shift. In line with this classical model, the results showed that, in young adults, the increase in the peak of centre of pressure shift towards the swing leg side in the RT condition was associated with a greater peak of propulsive forces towards the stance leg side. Because the propulsive forces were applied to the centre of gravity during a shorter time in the RT condition than in the SI condition, the participants were able to reach an equivalent centre of gravity dynamic at foot-off. Therefore, these results suggest that, in the RT condition, the young adults adapted the amplitude of the ML centre of pressure displacement to the shorter APA duration.

Like the young adults, the elderly adults were able to shorten APA duration in the RT condition (this shortening reached up to 38%). But, in marked contrast with the young adults, the elderly adults did not compensate for this APA shortening by increasing their peak of ML centre of pressure displacement. The mean peak value even tended to reach a lower value in the RT condition (3.4 cm) than in the SI condition (4.0 cm) (no significant difference). It follows that, according to the classic model of centre of gravity/centre of pressure interaction depicted above [26], [27], the anticipatory peak of propulsive forces (or, equivalently, the peak of centre of gravity acceleration) towards the stance leg side might *a priori* be expected to remain unchanged or even be reduced in the RT condition. Because the propulsive forces are applied to the centre of gravity within a much shorter time in the RT condition, postural dynamics at swing foot-off time might consequently be reduced, with the risk that this dynamic becomes insufficient to propel the centre of gravity above the base of support. However, in marked contrast with these expectations, the results showed that, as in young adults, the peak of centre of gravity acceleration in the elderly adults reached a greater value in the RT condition than in the SI condition. In addition, all indicators of postural dynamics at foot-off remained unchanged. These results show that, like the young adults, the elderly adults were able to adapt the amplitude of the propulsive forces to the shortening of APA duration. 

The adaptive strategy used by the elderly adults to maintain equivalent postural dynamics at foot-off was, however, different from the young adults, i.e. it did not imply an increased centre of pressure shift as expected from the classic model [26,27]. This observation is congruent with a recent study [35] which compared ML stability control during step initiation among young and older adults. The authors noticed a lack of significant age-related differences in the peak lateral displacement of the centre of pressure, despite an increased centre of gravity displacement towards the stance limb among the older adults. Therefore, a centre of pressure shift does not seem to be the sole strategy for accelerating the centre of gravity during APA in older adults. In the present study, the acceleration of body segments towards the stance leg side, e.g. with a slight trunk inclination around the hip and/or controlateral arm abduction [19,36] likely contributed to further increase centre of gravity acceleration in the RT condition. For example, previous studies have shown that displacing the arm forward during gait initiation might increase forward centre of gravity acceleration by as much as 30% as compared to isolated stepping, without any increase in anticipatory centre of pressure shift or APA duration [17,18]. Such arm and trunk strategies have been reported to be frequently employed by older adults when the centre of gravity has to be moved quickly [37,38], as is the case in the RT condition. In the present study, this strategy might compensate for the inability of elderly adults to further increase the peak of centre of pressure displacement during APA, probably due to weakness in the lower limb muscles. 

### The influence of temporal pressure on postural dynamics at swing foot-off in young and elderly adults

The young adults showed negative STAB1 values, indicating that the centre of gravity had not yet passed above the centre of pressure at foot-off. The centre of gravity velocity at this time was positive, i.e. it acted to bring the centre of gravity towards the new base of support. The results further showed that STAB2 was virtually zero in the young adults (in both the RT and SI conditions), which suggested that the postural dynamics at this time were precisely tuned to propel the centre of gravity above the centre of pressure position at foot-off. According to the concept of “extrapolated centre of mass” [29], this scenario implies that no action on the centre of pressure will be needed to reach a stable final posture. Contrary to the young adults, the elderly adults produced virtually null STAB1 value, indicating that the centre of gravity was positioned nearly above the centre of pressure at foot-off. As for the young adults, the centre of gravity velocity at this time reached a peak value toward the stance leg side. It follows that the postural dynamics generated at foot-off acted to propel the centre of gravity beyond the centre of pressure position at foot-off, i.e. toward the external side of the stance foot. The calculation of the STAB2 variable confirmed this trend and indicated that, with the actual postural dynamics at foot-off, the centre of gravity is expected to be propelled 3.3 cm beyond the centre of pressure position at foot-off (in both the RT and SI conditions), i.e. closer to the limits of the base of support as compared to the young adults. An adjustment of the centre of pressure position toward the external side of the stance foot will then be required to reach a stable final posture [29]. It is worth noting that such a strategy increases the risk of lateral imbalance, particularly when the centre of gravity is propelled to the extreme limit of (or beyond) the base of support. Then, a cross-over step might be required to recover balance, although this strategy is known to create a high risk of falling [39]. In this context of increased risk of lateral imbalance, it seems particularly important for elderly adults to shorten APA duration in the RT condition, since the increase in the peak of ML propulsive forces exacerbates the risk of lateral imbalance. This risk coping strategy might explain why the relative decrease in APA duration with temporal pressure was greater in the elderly adults (38%) than in the young adults (28%). 

## Conclusions

This work examined the aging-related effects of temporal pressure on two aspects of motor coordination, i.e. synchronization between two voluntary movements, and coordination between posture and movement. On the first aspect, the results showed that Paillard’s model of temporal coordination can be extended to paired movements involving the whole body. The results further showed that the ability to adequately synchronize the focal motor commands according to temporal pressure was preserved in the elderly, despite greater synchronization error and lower synchronization stability found in the elderly adults (in the SI condition only). Although proprioceptive inflow from the active limbs is known to be altered with aging, it seems to remain a sufficiently reliable source of information for movement synchronization. On the second aspect, the results showed that both the young and the elderly adults were able to increase their centre of gravity acceleration when the APA duration was drastically shortened as in the case of the situation with high temporal pressure. The adaptive strategy used to increase these propulsive forces, however, changed markedly with aging, and might expose older adults to a higher risk of lateral imbalance and falling. Thus, the present study provided new insights into the ability of older adults to adapt the postural and focal component of a paired movement involving the whole body to a temporal pressure constraint. These results might improve the understanding of the mechanisms contributing to the increased risk of falls in older adults. 
